# Severe pressure injury secondary to internet gaming addiction: a rare diagnosis in a neurologically intact patient

**DOI:** 10.1093/jscr/rjaf488

**Published:** 2025-07-08

**Authors:** Emily R Moran, Colin Song

**Affiliations:** Department of Plastic and Reconstructive Surgery, Fiona Stanley Hospital, Murdoch 6150, Australia; Department of Plastic and Reconstructive Surgery, Fiona Stanley Hospital, Murdoch 6150, Australia

**Keywords:** pressure injury, internet gaming disorder

## Abstract

Pressure injuries are preventable ischaemic injures that occur in regions of sustained pressure or shearing force, leading to localized tissue hypoxia and necrosis. These injuries do not occur commonly in people with intact neurological function including normal sensation and mobility. This report documents a case of stage 4 pressure injuries in a neurologically intact young man, secondary to Internet Gaming Disorder (IGD). This addiction to gaming is associated with many of the pre-disposing factors for pressure injury development, including immobility and poor tissue health secondary to nutritional deficiencies. This case details a multi-disciplinary team approach with substantial psychiatric input. IGD is a key area of future research within mental health literature, and this report documents one of many sequelae of this addiction.

## Introduction

Pressure injuries (PI) are preventable ischaemic injures that occur in regions of sustained pressure or shearing force, leading to tissue hypoxia and necrosis. The mechanism by which this occurs is commonly understood as external compression forces exceeding that of local capillary arterial pressure, resulting in an area of hypoxic tissue [1]. Pressure injuries are a significant healthcare problem and cost burden in Australia [2]. Well recognized populations at risk of PI include those with impaired mobility or sensation, cognitive impairment, sedation, immobilization, or hospitalized patients [3]. This case report presents the first published case of grade 4 sacral and ischial pressure injury in an otherwise sensate and fully mobile individual, secondary to Internet Gaming Disorder (IGD).

## Case report

A 34-year-old man presented to the emergency department due to worsening wounds at his sacral region. The patient reported awareness of these wounds developing, including increasing pain whilst sitting. Despite this pain he reported spending greater than 12 h per day sitting with direct pressure on the wounds whilst internet gaming. He reported occasional rigours. He reports utilizing opioid analgesia for the pain associated with the wound development and utilizes an indwelling catheter to negate the need for movement to the bathroom. He reports poor intake, utilizing energy drinks as his main source of nutrition.

On presentation he had a low-grade tachycardia and was afebrile. He appeared pale and malnourished, with reduced muscle tone. Examination of the sacral area demonstrated two wounds consistent with pressure injuries, a sacral wound of 4.5 × 2 cm, 2 cm deep with sacral bone on show at the base of the wound and a left ischial pressure wound of 6 × 5 cm, 4.5 cm deep with purulent discharge. Neurological examination, sensation and gait were normal.

**Figure 1:**
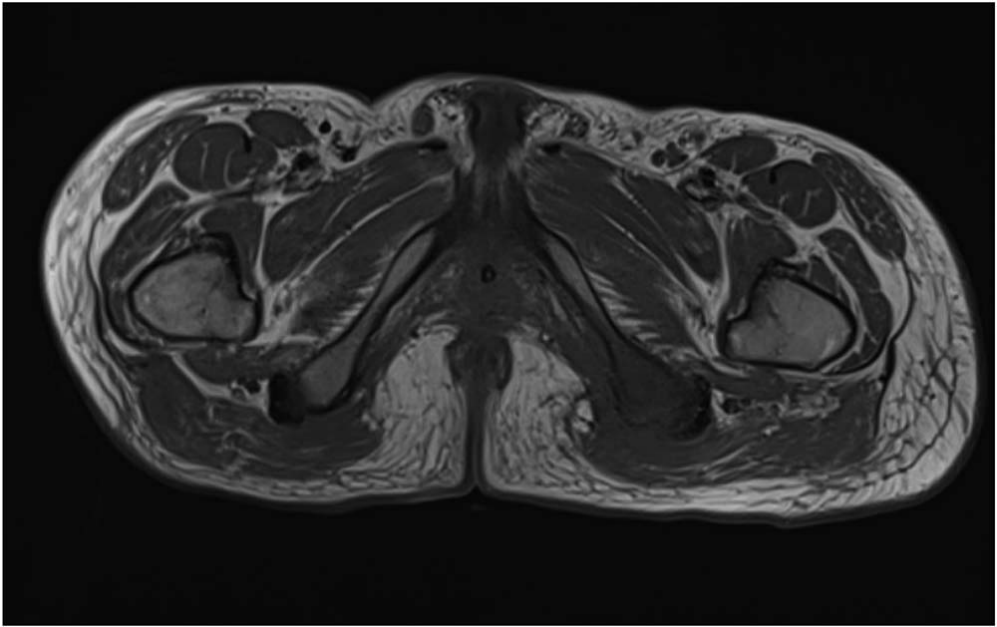
Axial MRI image demonstrating loss of normal T1 bone marrow signal is demonstrated involving the left ischial tuberosity. Findings in keeping with osteomyelitis.

**Figure 2:**
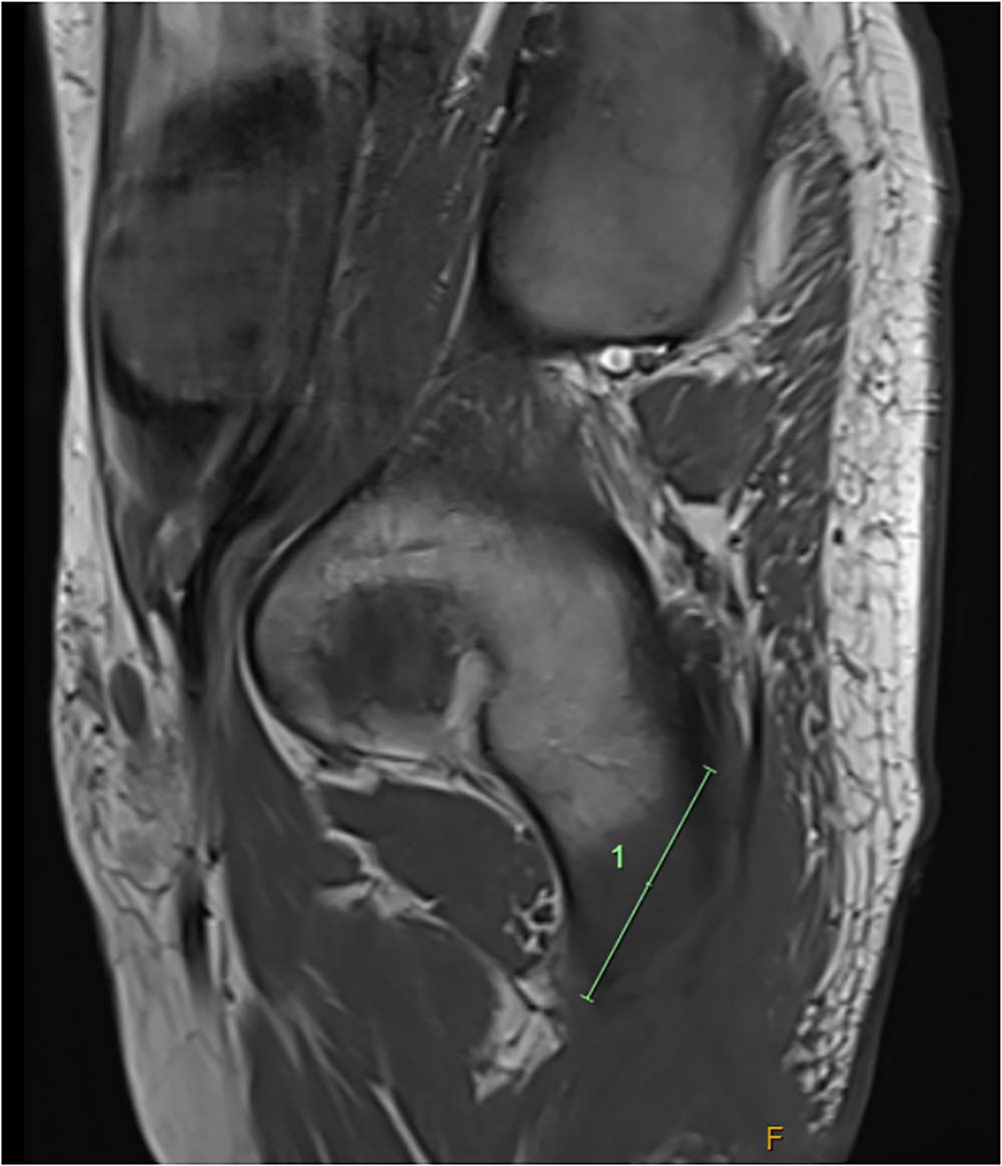
Sagittal MRI imaging demonstrating bony signal changes extend to involve the inferior pubic ramus medially.

He was admitted to the Plastic and Reconstructive Surgery service for investigation and multidisciplinary management. Initial blood tests demonstrated mild anaemia, mildly elevated white cell count. His C reactive protein was 39. Liver and renal function tests were normal. Magnetic resonance imaging (MRI) demonstrated features of osteomyelitis within the left ischial tuberosity (Fig. 1) and bony involvement extending to the inferior pubic ramus (Fig. 2). Surgical biopsy was performed, with confirmation of osteomyelitis and deep tissue infection. Growth of staphylococcus epidermis and streptococcus anginous was demonstrated in pressure wound and bone chip samples.

Given the complex psychosocial issues surrounding the development of grade 4 sacral and ischial pressure wounds in this patient, a multidisciplinary approach was required. Involvement of allied health teams including occupational health and physiotherapy input was required for strict offloading management. Ongoing specialist wound care nurse management, dietician review, infectious disease team guidance of ongoing antimicrobial therapy and acute pain service review for reduction in opioid reliance were involved throughout admission.

Pressure offloading compliance was challenging secondary to the patient’s preference for a seated position whilst internet gaming. Involvement of a hospital-based specialist Gaming Addiction Service was sought, and diagnosis of IGD was made. Identification of IGD allowed for treatment of predisposing factors such as depression and substance abuse. With clinical improvement in wound healing identified, the patient was discharged to outpatient management including regular clinical reviews and specialist community wound care nursing.

From the time of discharge this patient experienced a relapsing and remitting course of wound breakdown and improvement, with eventual wound healing. He continues to be actively involved in psychiatric follow up for IGD. Ongoing psychiatric and pain service involvement as an outpatient will form the foundation for future pressure wound prevention, through improvement in mental health factors and engagement with health behaviours such as pressure area care.

## Discussion

Pressure injuries develop from a complex interplay of patient and environmental factors, in the setting of prolonged exposure of soft tissues to sustained mechanical loading such as compression, tension or shear [4]. Patient risk factors for PI development include tissue factors and mobility factors. To our knowledge this is the first reported case of pressure injury development secondary to IGD, in an otherwise cognitively and neurologically intact patient. Pressure injury development is very rare in patients with intact mobility and sensation due to the natural inclination to reposition throughout the day secondary to discomfort from prolonged pressure [4].

IGD is recognized as a disorder of addiction within the International Classification of Diseases [5] and is listed as a topic for further research in the Diagnostic and Statistical Manual of mental disorders [6]. Due to the emerging nature of this disorder studies documenting the physical consequences of this disorder are limited [7]. Research by Benchebra *et al*. [7] reported an increased association with poor sleep, gastrointestinal dysfunction and cardiovascular disease in participants diagnosed with IGD. Additionally, Byeon *et al*. [8] found an association between those at risk of developing IGD and symptoms of dry eyes, impaired sleep, reduced physical activity, and increased risk of near-miss accidents.

Pressure injuries in sensate individuals with non-impaired mobility are exceedingly rare. In the case of this patient, the responsibility of pressure offloading is his own, as he has intact cognition, mobility and sensation to the affected area. As the foundation of PI management is pressure offloading, a multidisciplinary approach is crucial to treat both the PI itself and the confounding and exacerbating factors such as mental health disorders, opioid misuse and malnutrition. This case presents an example of grade 4 pressure injury secondary to IGD successfully treated with a combination of inpatient and outpatient wound care management, and involvement from a multidisciplinary team. It also highlights the importance of diagnosis of IGD, to allow for speciality team involvement and access to services to address underlying psychiatric comorbidities and predisposing factors.
